# Supplementary material for: Allium-Derived Compound Propyl Propane Thiosulfonate (PTSO) Attenuates Metabolic Alterations in Mice Fed a High-Fat Diet through Its Anti-Inflammatory and Prebiotic Properties
Source: Nutrients. 2021 Jul 28;13(8):2595. doi: 10.3390/nu13082595 (PMC8400710; doi:10.3390/nu13082595)
Supplement: Supplementary file 1 [file nutrients-13-02595-s001.zip › nutrients-1316644-supplementary.pdf]

## Materials and methods

### Reagents

PTSO (93% purity) has been developed, isolated and provided by DOMCA S.A. (Granada, Spain). Diets were provided by Scientific Animal Food & Engineering (Augy, France). All the other reagents have been purchased from Merck Life Science S.L.U. (Madrid, Spain), unless another provider is specified.

### Glucose tolerance test and plasma determinations

50% glucose solution in water was administered intraperitoneally (2 g/kg) and a blood sample was obtained from the tail vein at 0, 15, 30, 60 and 120 min. Glucose was measured with a Contour Next glucometer (Ascensia, Barcelona, Spain).

Glucose and cholesterol were measured by colorimetric methods using Spinreact kits (Spinreact, S.A., Girona, Spain). Plasma insulin was quantified by ELISA (Alpco Diagnosis, Salem, MA, USA), following the instructions provided by the manufacturers.

The Insulin Resistance Index was calculated according to the homeostatic model (HOMA-IR) and plasmatic lipopolysaccharide (LPS) levels were quantified by the Pierce™ Chromogenic Endotoxin Quant Kit (Thermo Scientific, Inc., Waltham, MA, USA).

### NADPH oxidase activity

The aortic rings were incubated for 30 minutes at 37°C in HEPES-containing physiological salt solution (pH 7.4) Aortic $O_2$- production was stimulated by addition of NADPH (100 µM). Rings were then placed in tubes containing physiological salt solution, with or without NADPH, and lucigenin was injected automatically at a final concentration of 5 µmol/L to avoid known artifacts when used at higher concentration. NADPH oxidase activity were determined by measuring luminescence over 200 s in a scintillation counter (Lumat LB 9507, Berthold, Germany) in 5-s intervals and was calculated by subtracting the basal values from those in the presence of NADPH. Vessels were then dried, and dry weight was determined. NADPH oxidase activity is expressed as relative luminescence units (RLU)/min/mg dry aortic ring.

**Flow cytometry**

Both tissues were isolated excised, washed and cut into small pieces (≤ 3 mm3) using sterile scissors or scalpel blades. The fat minced tissue was resuspended in a HBSS solution containing 1 mg/mL collagenase Type I. These were transferred into a 50 mL tube and placed in a 37 °C water bath for 30 min. After that, the digested tissue solution was filtered using 100 μm cell strainers. The pellet was resuspended in HBSS to wash it before passing it through a 70 μm cell strainer. Then, the red blood cells were removed using a red blood cell lysis buffer for 5 minutes at room temperature. Cells were centrifuged and the pellet was resuspended in HBSS medium to wash it. The harvested cells were stained for different markers (Miltenyi Biotec GmbH, Bergisch Gladbach, Germany), unless otherwise stated. $2 \times 10^6$ cells were counted and submitted to FcγR blocking. After that, the cells were stained in the surface with Viability Dye (eFluor 780, REF. 65-0865-14; eBioscience, San Diego, USA), anti-CD3 (AmCyan, Clone 145-2C11), anti-CD4 (APC-Vio 770, Clone REA1211), anti-CD11b (APC-Vio 770, Clone REA592), anti-CD11c (FITC, Clone N418) and LyC6 (PE-Vio770, Clone 1G7.G10) for 15 min at 4 °C in the dark. The cells were then fixed with the Flow Cytometry Fixation Buffer (R&D Systems®, Catalog #FC004) Data collection was performed using a flow cytometer CANTO II (BD Biosciences, New Jersey, USA) and analyzed by Flowjo (Flowjo LLC, Ashland, Oregon, USA).

**Tables**

**Table S1.** qPCR primer sequences.

| Gene | Organism | Sequence 5'- 3' | Annealing T °C |
|---|---|---|---|
| ***Adiponectin*** | Mouse | FW: GCGAGAGTTTCTGGCAGAGT<br>RV: CCGAGATAGCTGCCAGAGTT | 60 |
| ***Ampk*** | Mouse | FW: GACTTCCTTCACAGCCTCATC<br>RV: CGCGCGACTATCAAAGACATACG | 60 |
| ***Gapdh*** | Mouse | FW: CCATCACCATCTTCCAGGAG<br>RV: CCTGCTTCACCACCTTCTTG | 60 |
| ***Glut 4*** | Mouse | FW: GAGAATACAGCTAGGACCAGTG<br>RV: TCTTATTGCAGCAGCGCCTGAG | 62 |
| ***Il-1β*** | Mouse | FW: TGATGAGAATGACCTCTTCT<br>RV: CTTCTTCAAAGATGAAGGAAA | 60 |
| ***Il-6*** | Mouse | FW: TAGTCCTTCCTACCCCAATTTCC<br>RV: TTGGTCCTTAGCCACTCCTTCC | 60 |

| | | | |
|---|---|---|---|
| ***Jnk-1*** | Mouse | FW: GATTTTGGACTGGCGAGGACT<br>RV: TAGCCCATGCCGAGAATGA | 60 |
| ***Jnk-2*** | Mouse | FW: TTGTGCTGCTTTTGATACAGTTCTTGGG<br>RV: CTGGAAAGAGCTCTTCAAATTTGAT | 60 |
| ***Leptin*** | Mouse | FW: AGATCCCAGGGAGGAAAATG<br>RV: TGAAGCCCAGGAATGAAGT | 60 |
| ***Leptin R*** | Mouse | FW: GCTATTTTGGGAAGATGT<br>RV: TGCCTGGGCCTCTATCTC | 60 |
| ***Muc-2*** | Mouse | FW: GCAGTCCTCAGTGGCACCTC<br>RV: CACCGTGGGGCTACTGGAGAG | 60 |
| ***Muc-3*** | Mouse | FW: CGTGGTCAACTGCGAGAATGG<br>RV: CGGCTCTATCTCTACGCTCTCC | 60 |
| ***Occludin*** | Mouse | FW: ACGGACCCTGACCACTATGA<br>RV: TCAGCAGCAGCCATGTACTC | 56 |
| ***Ppar- α*** | Mouse | FW: TCGAGGAAGGCACTACACCT<br>RV: TCTTCCCAAAGCTCCTTCAA | 60 |
| ***Ppar-γ*** | Mouse | FW: ACGATCTGCCTGAGGTCTGT<br>RV: CATCGAGGACATCCAAGACA | 60 |
| ***Tlr4*** | Mouse | FW: GCCTTTCAGGGAATTAAGCTCC<br>RV: AGATCAACCGATGGACGTGTAA | 60 |
| ***Tnf-α*** | Mouse | FW: AACTAGTGGTGCCAGCCGAT<br>RV: CTTCACAGAGCAATGACTCC | 60 |
| ***Zo-1*** | Mouse | FW: GGGGCCTACACTGATCAAGA<br>RV: TGGAGATGAGGCTTCTGCTT | 56 |

**Figures**

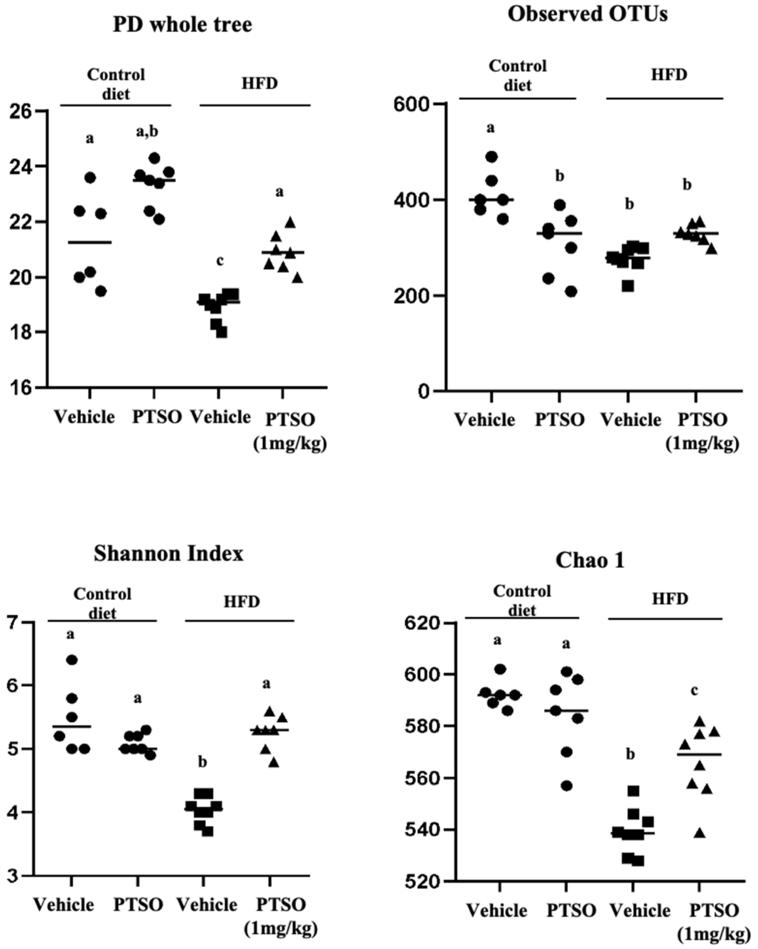


**Figure S1.** Impact of PTSO administration on microbiome diversity (PD whole tree, Observed OTUs, Shannon index and Chao1). Groups with different letters statistically differ (P<0.05).
